# Proceedings of the 26th International Analytical Ultracentrifugation Workshop and Symposium

**DOI:** 10.1007/s00249-025-01799-z

**Published:** 2025-09-27

**Authors:** Johannes Walter, Alexander Bepperling

**Affiliations:** 1 Institute of Interfaces and Particle Technology (IPT), Friedrich-Alexander-Universität Erlangen-Nürnberg (FAU), Cauerstraße 4, 91058 Erlangen, Germany; 2 Interdisciplinary Center for Functional Particle Systems (FPS), Friedrich-Alexander-Universität Erlangen-Nürnberg (FAU), Haberstraße 9a, 91058 Erlangen, Germany; 3 Biodevelopment/Sandoz Global Development, Hexal AG, Industriestraße 18, Building 2, 83607 Holzkirchen, Germany

## Abstract

The 26th International Analytical Ultracentrifugation Workshop and Symposium (AUC2024) took place at the scenic Banz Abbey near Bad Staffelstein, Germany, from July 22 to 27, 2024. 84 participants from 16 countries (Belgium 1, Canada 7, China 3, Colombia 1, Czech Republic 3, Finland 1, France 3, Germany 35, Israel 1, Italy 1, Japan 2, New Zealand 2, Spain 1, Switzerland 4, United Kingdom 5, United States 14) travelled to Germany to present and discuss the latest advances in the field. 40 workshop sessions were held by world-leading experts covering all aspects of AUC including experimental design, data analysis and data processing according to good manufacturing practice (GMP), but also complementary methods such as hydrodynamic modelling, isothermal titration calorimetry and small angle X-ray scattering data processing were considered. A visit to a stalactite cave in Franconian Switzerland and the Bavarian beer museum in Kulmbach offered a welcome change to the scientific program and started off the 3-day symposium. The presentations featured of course AUC, but also dynamic and static light scattering, small angle X-ray and neutron scattering, surface plasmon resonance, field flow fractionation, calorimetry, chromatography, and electron microscopy. The AUC2024 special volume provides a comprehensive overview of the sustained innovation, utility and relevance of AUC and related solution biophysical and particle technology methods across various disciplines, including biochemistry, structural biology, synthetic polymer chemistry, carbohydrate chemistry, protein and nucleic acid characterization, nano and colloids science, and macromolecular interactions.

## Conference organization

During the 25th International Analytical Ultracentrifugation Workshop and Symposium in Lethbridge, Canada, it was decided to hold the next meeting in Germany hosted by Johannes Walter (FAU Erlangen-Nürnberg, Germany) and Alexander Bepperling (Sandoz, Germany). They were thrilled to bring this meeting back to Germany after almost 20 years.

We would like to sincerely thank the scientific organizing committee (Olwyn Byron (University of Glasgow, United Kingdom), Borries Demeler (University of Lethbridge, Canada), Karen Fleming (Johns Hopkins University, USA), Trushar Patel (University of Lethbridge, Canada), Klaus Richter (Coriolis Pharma, Germany), Christine Ebel (Univ. Grenoble Alpes, France) and Susumu Uchiyama (Osaka University, Japan)) for fruitful discussions during the preparation of the meeting, especially around the Svedberg award. We also like to express our gratitude to the local organization committee (Ina Viebach, Michael Hartmann, Angelika Mach, Paola Cardenas Lopez, Lisa Stiegler and Moritz Moß (all from FAU Erlangen-Nürnberg, Germany). Their dedication and organizational talent made this conference a success.

Traditionally, the event started with 2 days of workshops presented by world-leading experts, often the creators of software analysis packages such as UltraScan, SedAnal, Sedfit, DCDT +, Sednterp, SVEDBERG, Sedview, GUSSI, SViMULATE, SOMO, HDR-MULTIFIT and HDR-SVFIT. The workshops illustrated the extreme versatility of AUC including the analysis of nanoparticles, macromolecules and colloidal systems, reversible interactions of biological systems, gene therapy vectors, but also hydrodynamic modelling and the analysis of glyco- and membrane proteins. A novelty this year was the demonstration of GMP-compliant AUC analysis using software such as BASIS and UltraScan GMP, which became relevant due to the rise of adeno-associated viruses (AAVs) as therapeutic agents. Data analysis of complementary techniques such as mesoscale thermophoresis, biolayer interferometry, isothermal titration calorimetry, small angle X-ray and neutron scattering as well as fluorescence spectroscopy was also covered. The mixture of hands-on training and in-depth discussion of analytical and software concepts made the workshops valuable for beginners as well as for experienced scientists.

The trend of strong in-person participation by the younger generation was encouraging (see Table 1). Students and postdoctoral fellows turned out in greater numbers for oral and poster presentations than at previous conferences. In-person participation by female scientists also increased slightly compared to previous conferences, although there is still room for improvement.

**Table 1:** Metrics for gender and career stage in-person participation

	Gender		Career stage			
	Female	Male	Student	Postdoc	Established	Industry
Registrations	32% (27)	68% (57)	25	16	25	18
Session chairs	36% (4)	64% (7)	0	2	8	1
Talks	30% (13)	70% (30)	8	10	21	4

## Social event

**Fig. 1:**
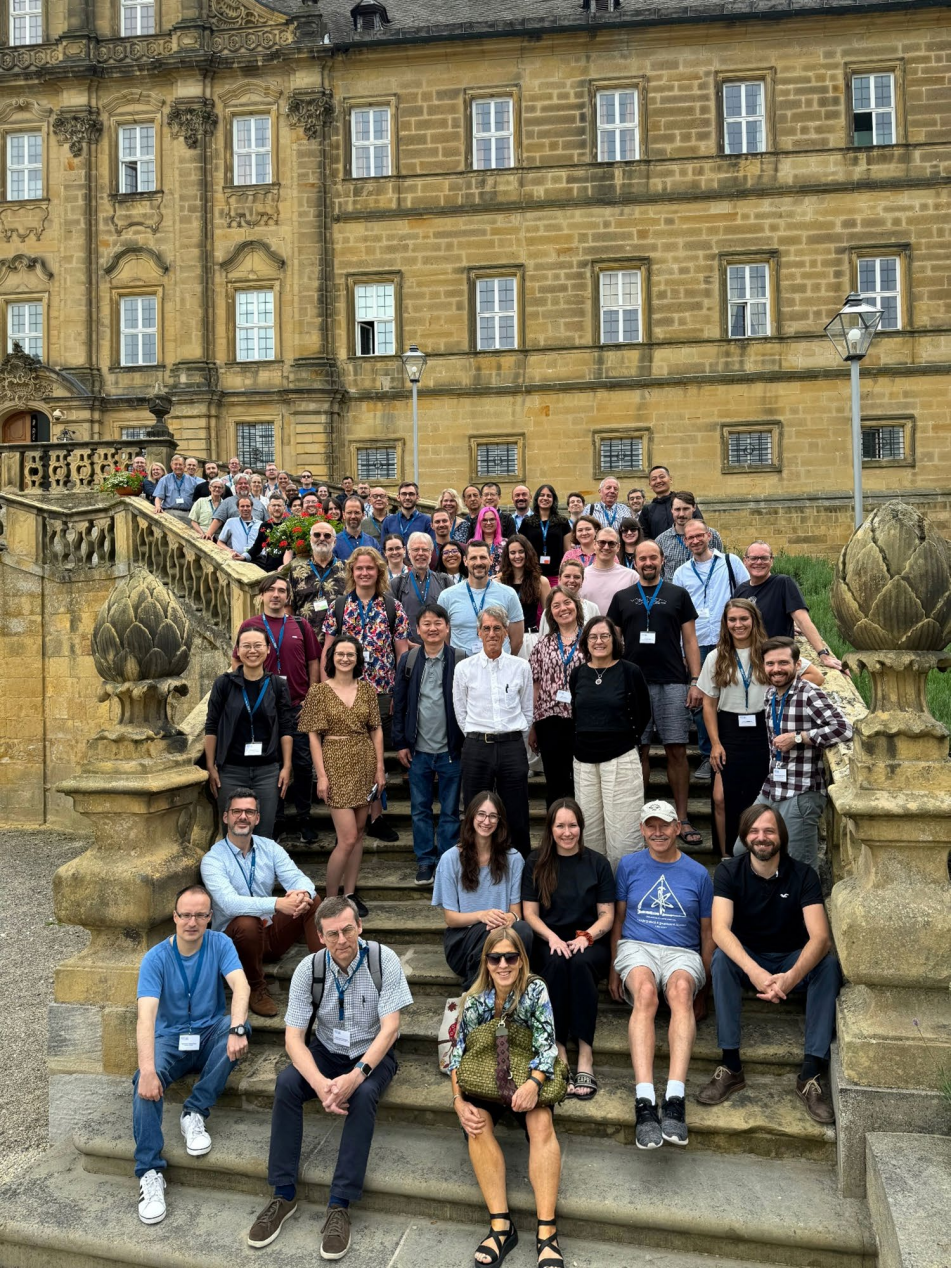
Group shot in front of Banz Abbey

The participants enjoyed a tour to Rabenstein Castle and the Sophie Cave and concluded the day with a stop at the Bavarian Brewery Museum in Kulmbach and the conference dinner at our venue site at Banz Abbey (Fig. 1).

## Svedberg award

The award honours individuals who have made significant contributions to the field of AUC and hydrodynamics. These contributions include the development of software, theoretical frameworks, instrumentation or the spread of the technology. This year, the scientific organizing committee elected Helmut Cölfen posthumously for his outstanding contribution and his life-long dedication to the field of polymer and colloid sciences.

In memory of his passion towards teaching and his dedication for his students, the committee also decided to launch the Cölfen Early Career Investigator Award (CECIA). It was awarded to Ivo Nischang (University of Jena, Germany) for his work on polymer characterization by AUC.

## The proceedings of the 26th International Analytical Ultracentrifugation Workshop and Symposium

The AUC2024 proceedings contain 10 articles covering the latest developments in the field of AUC and hydrodynamics.

### Software development and improvements

Band-forming experiments use layered solutions of different densities in AUC, enabling the study of diverse systems. However, they are rarely applied because existing software cannot properly account for density and viscosity changes caused by diffusive mixing. To address this, Dobler et al. developed and validated a predictive model that captures these dynamic changes, improving the interpretation of band-forming experiments and highlighting the need for updated analysis tools (Dobler et al. 2025).

Simulating sedimentation velocity AUC (SV-AUC) data provides valuable support for experimental planning and hypothesis testing. Brautigam developed the *SViMULATE* software to simplify the simulation of SV-AUC data, providing an intuitive interface and automatic conversion of macromolecular properties into key hydrodynamic parameters (Brautigam 2025). Since its initial release (Brautigam 2023), the program has been expanded to support additional experimental modes such as interacting systems, nonideal sedimentation, floatation, and band SV. Version 1.4.0 also introduces improved numerical stability, polydisperse species modelling, and advanced discretizations, greatly broadening its scope and utility.

Peter Schuck’s analysis software *SEDFIT* enjoys wide popularity in the biopharmaceutical industry for the determination of trace aggregates. The calculation of confidence limits for their relative concentration and sedimentation coefficient is crucial for a solid risk assessment of these minor components. John Philo developed a new method to calculate these confidence limits and implemented it in the latest version (7.0) of his software *SVEDBERG* (Philo 2025). The procedure involves the conversion of a continuous c(s) distribution into a discrete species model and the definition of a “master” species, usually the main species, which subsequently defines the molar mass for all other species. After optimization of its hydrodynamic parameters, the user can proceed to calculate the confidence limits for the relative abundance and S-values of up to six species with concentrations of far less than 1%.

Hydrodynamic modelling and the computation of hydrodynamic parameters such as sedimentation and diffusion coefficients from structural models are nowadays offered as user-friendly software packages requiring little prior knowledge. Nevertheless, the user should strive for an understanding of the fundamental theory behind those computations for an accurate interpretation of the results as de la Torre and Hernández-Cifre point out in their assay on classical and modern concepts of macromolecular hydrodynamics (de la Torre and Hernández-Cifre 2025).

A systematic comparison of two-bead modelling approaches revealed subtle but consistent differences for the computation of hydrodynamic properties of macromolecules such as the translational diffusion coefficient and the intrinsic viscosity (Brookes et al. 2025). Furthermore, the authors have developed a set of correction functions to match the results of the faster “one bead per residue” with the computational more intensive “one bead per atom” approach. Both methods are now implemented in the software suite *US-SOMO*.

### Biological applications

Free antibody light chains (FLCs) found in serum, cerebrospinal and synovial fluid and urine are a hallmark of certain pathological conditions, often associated with blood cancer such as multiple myeloma. Their fibrillation and deposition in the kidney can lead to severe organ damage. Tucholsky and colleagues investigated the association state of FLCs isolated from urine of nine multiple myeloma patients under native and reducing conditions using SV-AUC, Far-UV-CD spectroscopy and differential scanning fluorometry (Tucholski et al. 2025). They found a time-dependent increase of fragmentation and aggregations upon reduction for all nine patients with considerable variation amongst them.

Since their first description in 2001 (Sakamoto et al. 2001), PROTACs (Proteolysis Targeting Chimeras) have opened a new way of regulating protein activity via small molecular drugs. Unlike conventional small molecular drugs, which aim to regulate the protein activity by inhibition or activation, these bifunctional molecules hijack the cellular protein degradation machinery by enforcing binding between the target protein and the E3 ubiquitin ligase and thus sending the target protein down the degradation pathway. Yarawsky and his colleagues rebrand the often overlooked SV-AUC experiment as a multi-attribute platform method for the in-depth analysis of targeted protein degradation (Yarawsky et al. 2025). Studying the interaction between Bruton’s tyrosine kinase (BTK) and Cereblon (CRBN), they were able to assess sample purity, percent ternary complex, binding and kinetic rate constants, and hydrodynamics in a single SV-AUC experiment. Sedimentation equilibrium AUC can further complement the SV-AUC data to confirm stoichiometry.

The world’s most abundant enzyme RUBISCO (Ribulose-1,5-Bisphosphate carboxylase/oxygenase) needs to be activated by a specific activase, Rubisco activase (Rca). Unlike most AAA + proteins, Rcas do not form hexamers in solution but exhibit a high degree of polydispersity. Keown and co-workers have isolated the Rca α- and β-isoforms of cotton, creosote, Antarctic hairgrass, and spruce Rca and investigated their oligomerization behaviour and thermal stability (Keown et al. 2025). They found significant diversity in the oligomeric assembly, thermal stability, and functional activity of Rca isoforms, revealing species-specific adaptations that reflect their ecological niches.

### Macromolecular assemblies

The rise of AAVs as biomedicines helped to spread analytical and preparative ultracentrifugation in the biopharma field. Caesium chloride density gradient ultracentrifugation (CsCl-DGUC) is still considered as the gold standard for the large-scale purification of AAVs. The heterogeneity of the “full” fraction remains a challenge. Hirohata et al. have elaborated how the presence of DNA in the full capsids amplifies the difference in the partial specific volume caused by different stoichiometries of the three-capsid proteins leading to a broad density distribution in the CsCl-gradient (Hirohata et al. 2025).

A fascinating crossover of protein and inorganic chemistry was presented by Fakhouri and co-workers using SV-AUC (Fakhouri et al. 2025). A certain species of metallodendrimer, cobaltabisdicarbollide (COSAN), self-assembles into micelles and thereby promotes protein aggregation in a controlled fashion. The authors have unravelled the stoichiometry and the aggregation pathway of the COSAN-induced formation of myoglobin-COSAN assemblies.

## Conclusion

The 26th International Analytical Ultracentrifugation Workshop and Symposium (AUC2024) was a huge success. More than 80 participants from all over the world enjoyed workshops presented by world-leading experts. They presented their own work and enjoyed lively discussions beyond the schedule. This proves the value of in-person conferences, not a given in the post-Covid world. Due to the generous support of Beckman Coulter, most of the oral presentations were professionally recorded and are available online: https://www.auc2024.fau.de/recordings/

Finally, we are looking forward to the next meeting hosted by Wenqi Li, Xiaodong Ye, Shaowei Li, and Mengdi Chen at Tsinghua University in Beijing in late August 2025. Please refer to https://www.frcbs.tsinghua.edu.cn/auc2025 for further information.

## Data Availability

There is no data availability statement available for this article.
